# Methotrexate-Related Liver Cirrhosis in Psoriatic Arthritis: A Case Report and Review of the Literature

**DOI:** 10.31138/mjr.32.3.264

**Published:** 2021-09-07

**Authors:** Maria-Loukia Koutsompina, Maria Pappa, Stratigoula Sakellariou, Chrysoula G. Gialouri, George E. Fragoulis, Theodoros Androutsakos

**Affiliations:** 1 Department of Pathophysiology, “Laiko” Hospital, School of Medicine, National and Kapodistrian University of Greece, Athens, Greece; 2 First Department of Internal Medicine, “Laiko” Hospital, School of Medicine, National and Kapodistrian University of Greece, Athens, Greece; 3 Department of Pathology, School of Medicine, National and Kapodistrian University of Greece, Athens, Greece

**Keywords:** Methotrexate, liver dysfunction, psoriatic arthritis, psoriasis

## Abstract

Methotrexate is an anchor-drug for the treatment of inflammatory arthritides affecting peripheral joints, such as rheumatoid and psoriatic arthritis (PsA), but also for other immune-mediated diseases like psoriasis. Although it is generally a well-tolerated drug, adverse effects often occur. Reversible derangement of liver function test is the most common laboratory adverse event. However, in some cases, liver cirrhosis and/or fibrosis can occur. Besides, many of these diseases like PsA and psoriasis are closely linked with clinical conditions and risk factors that also contribute to liver damage/cirrhosis, such as increased body mass index, dyslipidaemia and diabetes mellitus (DM). It has been hypothesised that the aforementioned risk factors along with methotrexate usage can act synergistically, causing liver damage in these patients. Herein, we describe a PsA patient with DM who developed fatal liver cirrhosis after 10 years of treatment with MTX. We also review the literature about the liver toxicity of MTX in the context of PsA and psoriasis, describing concurring risk factors and histopathological findings. PubMed and Scopus were searched, without date limits. The keywords “methotrexate” AND “psoriatic arthritis” OR “psoriasis” AND “Liver damage” OR “liver fibrosis” OR “cirrhosis” were used. We found that although fibrosis/cirrhosis is present in about 10–25% of the patients, MTX can rarely cause liver damage itself. However, it can exert its effect when other factors, like increased alcohol consumption and obesity coexist. Prospective studies are needed, specifically examining the hepatotoxicity of MTX in individuals with immune-mediated diseases.

## INTRODUCTION

Methotrexate (4-amino-10methylfolic acid, MTX), an antagonist of folic acid, is used widely in malignant and non-malignant diseases.^1,2^ It is currently used as first-line disease modifying anti-rheumatic drug (DMARDs) in the treatment of rheumatoid and psoriatic arthritis (PsA) as well as in inflammatory bowel disease and in other non-rheumatic disorders.^1–4^ MTX has a low cost and shows a good safety and efficacy profile. However, possible adverse events are always a concern.^1^

Abnormal liver blood tests in patients treated with MTX has long been an issue. Most medical associations suggest a baseline assessment of liver function tests before the introduction, followed by transaminases measurement initially every 1 or 2 weeks for the first month, and every 2–3 months subsequently. MTX discontinuation is advised when transaminase levels are greater than 3 times of the upper limit of normal (ULN).^5–7^ The exact mechanism of MTX-induced hepatotoxicity is debatable. It seems that formation of polyglutamated MTX metabolite and its prolonged retention in the hepatic cells plays a pivotal role.^8^ It has also been shown that MTX promotes adenosine release from hepatocytes. This, stimulate further production of collagen by hepatic stellate cell through adenosine A2a receptors, contributing thus to liver fibrosis.^9^

MTX-induced hepatotoxicity has been regarded as a major side effect in the previous decades. However, recent meta-analyses have shown that despite increased incidence of transaminasaemia among patients with inflammatory arthritis who receive MTX, this is predominantly asymptomatic and rarely leads to liver injury/fibrosis, cirrhosis, and treatment discontinuation.^10^ It is estimated that the prevalence of cirrhosis is low in patients without comorbidities even after long-term use.^11–14^ In contrast, when serious MTX-related hepatotoxicity or cirrhosis occurs, a second contributing factor is usually present like other hepatotoxic drugs, chronic hepatitis or alcoholic and non-alcoholic fatty liver disease, with the latter being the most common.^15,16^

Herein, we present a patient with psoriatic arthritis (PsA) and type 2 diabetes mellitus who presented to our hospital with cirrhosis accompanied by severe ascetic fluid accumulation. After excessive work-up that included a liver biopsy, a diagnosis of MTX-related cirrhosis was made. A literature review about this matter is also presented.

## CASE REPORT

A 65-year-old Caucasian male was admitted to our department due to large volume ascites. Three months before admission, the patient presented to another hospital with the same symptoms; a diagnosis of non-alcoholic steatohepatitis (NASH) induced cirrhosis was made and the patient was discharged with 40 mg furosemide and 75mg spironolactone daily. He had a medical history of PsA and type 2 diabetes mellitus, both diagnosed a decade ago, having normal levels of glycated haemoglobin (HbA1C) during the last five years. His medications included methotrexate once weekly being received for the last 6 years, with a cumulative dose of approximately 5gr, metformin and sitagliptin.

Upon presentation, he was afebrile, with a blood pressure of 105/80 mmHg, heart rate of 92 beats per minute and an oxygen saturation of 94%, on air. He had large volume ascites, lower extremities oedema, palpable liver and spleen, as well as spider nevi in the torso and palmar erythema. The remainder of the physical examination was normal. His laboratory tests showed an elevated bilirubin concentration (2,52mg/dl, direct: 1,21mg/dl) with mild increase of gamma glutamyl-transferase (γGT) (143 U/L) and alkaline phosphatase (ALP) (131U/L), normal levels of alanine (ALT) and aspartate (AST) aminotransferases and cholesterol, while international normalised ratio (INR) was 1,17 and serum albumin 2.8g/dl (**Table 1**). Additionally, no abnormalities in liver function tests including AST/ALT/ALP/γGT/INR and albumin were noted in blood results available before the initiation of MTX. Testing for hepatitis A, B and C, for anti-nuclear antibodies, anti-smooth muscle antibodies, and anti-mitochondrial antibodies was negative, while serum ceruloplasmin and urine copper levels were also within normal levels (**Table 1**).

**Table 1.** Patient’s laboratory findings on admission.

	**On admission**	**Reference Range**
Hgb (g/dl) / Hct (%)	13 / 38.4	13.5 – 18 / 40–50
WBC (K/*μL*)	8.28	4,5–11,0
Neutrophils/Lymphocytes/Monocytes (%)	71.2 / 15.1 / 11.8	
PLTs (K/*μL*)	172	140–440
PT / APTT (sec) / INR	13.9 / 33.9 / 1.17	12.4/ 29–40 / 1
Fibrinogen (mg/dL)	545	180–400
Glucose (mg/dL) /Glycosylated haemoglobin (%)	102 / 7.8	72–106 / 4.8–6
Urea / Creatinine (mg/dL)	42 / 1.1	40–60 / 0.7–1.2
Potassium / Sodium (mmol/L)	5.3 / 140	3.7–4.9 / 136–143
AST / ALT / GGT / ALP (U/L)	26 / 26/ 143/ 131	< 35 / <35 / 8–61/ 135–225
Total Bilirubin / Direct Bilirubin (mg/dl)	2.52 / 1.21	0.3–1.2 / 0.0–0.3
Amylase (U/L)	34	28–100
Cholesterol / Triglyceride (mg/dl)	163 / 99	140–200 /50–150
Lactate dehydrogenase (U/L)	341	135–225
Total protein / Albumin (g/dl)	6.6 / 2.8	6.0–7.9 / 3.5–5.0
Iron (*μg*/dL) / Ferritin (ng/dL)	108 / 325	38–190 / 30–400
B12 (pg/mL) / Folic acid (mg/mL)	731 / 7.5	223–925 / 3.9–27
CRP (mg/L)	43.43	0–5
TSH (mlU/L)	2.34	0.5 – 8.9
HBsAg / HBcAb / HCV Ab	- / - / -	- / - /-
ANA / ASMA / AMA	- / - /-	- /- /-

**Abbreviations:** Hgb: Haemoglobin; Hct: Haematocrit; WBC: White Blood Cells; PLTs: Platelets; PT: Prothrombin Time; APTT: activated partial thromboplastin time; INR: International Normalized Ratio; AST: Aspartate Aminotransferase; ALT: Alanine Aminotransferase; GGT: Gamma-Glutamyl Transferase; ALP: Alkaline Phosphatase; CRP: C-reactive protein; TSH: Thyroid Stimulating Hormone; HBsAg: Hepatitis B surface Antigen; HBcAb: Hepatitis B core Antibodies; HCV Ab: Hepatitis C Virus Antibodies; ANA: Anti-nuclear Antibodies; ASMA: Anti-Smooth Muscle Antibodies; AMA: Anti-Mitochondrial Antibodies.

**Figure 1.**
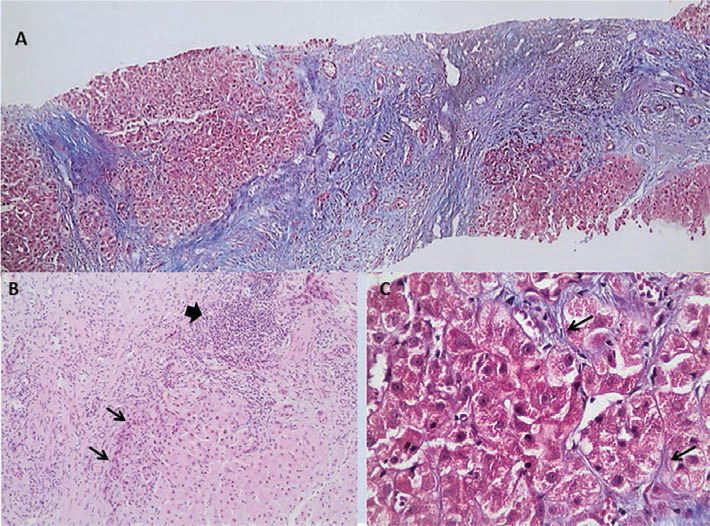
Liver biopsy shows micronodular cirrhosis. (A: Masson trichrome x40). Fibrous septa with lymphocytic infiltrates (arrowhead) and prominent ductular reaction (arrows) at the periphery of cirrhotic nodule (B: H/Ex200). Mild pericellular fibrosis (arrows) in a nodule (C: Masson trichrome x 400).

Ultrasonography of the abdomen revealed ascites, cirrhotic liver, splenomegaly, and a patent portal vein, while chest X-Ray showed right-sided pleural effusion. An ascites paracentesis was performed that revealed 383 cells, with 10% neutrophils and a serum albumin gradient (SAAG) of 2,5mg/dl, compatible with portal hypertension ascites (**Table 1**). Peritoneal fluid cytology and cultures were negative. Computed tomography (CT) of the abdomen revealed a cirrhotic liver with splenomegaly, portosystemic collaterals, and ascites. Magnetic Resonance Imaging (MRI) of the abdomen and Magnetic Resonance Cholangiopancreatography (MRCP) findings were in accordance with CTs’ findings; neither masses nor strictures in intra- or extrahepatic biliary ducts were identified. Upper gastrointestinal endoscopy was performed and revealed portal hypertensive gastropathy but no oesophageal or gastric varices. A transthoracic echocardiogram did not reveal heart failure, chronic compressive pericarditis, pathology of heart valves, or other heart disease that could lead to cirrhosis. Eventually, a liver biopsy was performed that revealed micronodular cirrhosis with lymphocytic infiltrates in fibrous septa and features of cholestasis at the periphery of cirrhotic nodules; no steatosis, ballooning, pericellular fibrosis, or interface hepatitis were noted (**Figure 1**).

According to clinical history and histological examination, end-stage liver disease was diagnosed, and MTX was discontinued. High doses of furosemide and spironolactone were introduced to control the accumulation of ascites, however patient developed renal failure. Subsequently, diuretics were withdrawn, and frequent large volume paracentesis were initiated. Unfortunately, the patient died 8 months after the initial diagnosis, due to spontaneous bacterial peritonitis.

## LITERATURE REVIEW

### Search strategy

A literature search for relevant articles using PubMed and Scopus was performed. No date limits were applied, and last update was in June 2020. The keywords “methotrexate” AND “psoriatic arthritis” OR “psoriasis” AND “Liver damage” OR “liver fibrosis” OR “cirrhosis” were used. Search fields were restricted in the “Title/abstract” and in human species. In total, 301 studies were retrieved. After removal of the duplicates, studies describing paediatric patients and non-English literature, 217 studies were available for extraction. From those only 31 observational studies were found. Reference lists of relevant articles were also reviewed.^17–47^

### Studies covering hepatotoxic effect of MTX. Data and pitfalls

Methotrexate has long been accused of severe hepatotoxicity, especially after long-term usage. However, the rising incidence of NASH-related cirrhosis and the association of psoriasis and PsA with the metabolic syndrome challenge the real hepatotoxic effect of methotrexate. As a matter of fact, a lot of studies have addressed this question in the literature, with the results being ambiguous. More precisely, most of the studies examining MTX-induced hepatotoxicity, yield a liver fibrosis ratio of 5.6 to 71 %, while cirrhosis ratio is even lower from 0 to 16.6% (**Table 2**). These studies have two major drawbacks; most of them were conducted 20 years ago, with 14 of them before 1990, and in only 5 of them liver biopsies (LB) before and after treatment are available.^39–41,43^ Thus, the net-effect of NASH in liver fibrosis cannot be safely assessed. Moreover, most of the studies include patients with psoriasis and only a few (n=6) patients with PsA.^20,24,26,27,33,43^

**Table 2.** Studies of methotrexate induced hepatotoxicity in psoriatic patients.

**First author, year (reference)**	**Number of patients**	**Disease**	**Type of study**	**Way of fibrosis assessment**	**Results**
Roenigk HH, 1971^17^	37	PsO	Prospective and Retrospective	LB	23 (62.1%) fibrosis 6 (16%) cirrhosis
Dahl MG, 1971^18^	37	PsO	Prospective	LB	10 (27%) fibrosis 7 (19%) cirrhosis
Millward-Sadler GH, 1974^29^	17	PsO	Retrospective	LB	4 (23.5%) fibrosis, 3 (17.6%) cirrhosis
Zacharie H, 1975^39^	56	PsO	Prospective	LB before and after treatment	18 (32.1%) fibrosis, 3 (5.3%) cirrhosis
Nyfors A, 1976^40^	88	PsO	Retrospective	LB before and after treatment	5 (5.6%) fibrosis, 6 (6.8%) cirrhosis after treatment
Zacharie H, 1980^41^	328 (764 LB)	PsO	Prospective	LB before and after treatment	19/183 (10.3%) cirrhosis in patients treated with methotrexate (25.6%) cirrhosis after 5-years of treatment with MTX
Ashton R, 1981^42^	38	PsO	Retrospective	LB	7 (18.5%) fibrosis, 2 (5%) cirrhosis
Lanse S, 1985^43^	30	PsO, PsA (n=18)	Prospective	LB before and after treatment	No worsening of LB when baseline LB normal 1/11 with fatty infiltration worsened after treatment
Van de Kerkhof PC, 1985^44^	44	PsO	Prospective	LB after treatment	9 (20.4%) fibrosis, 2 (4.5%) cirrhosis
Pestana A, 1985^45^	32	PsO	Retrospective	LB	15 (46.8%) fibrosis, 5 (15.6%) cirrhosis
Risteli J, 1988^19^	24	PsO	Prospective	LB	11 (45.8%) fibrosis, 4 (16.6%) cirrhosis
O’Connor G, 1989^37^	78 (95 LB)	PsO	Retrospective	LB	40/95 (42%) fibrosis
Newman M, 1989^47^	168 (364 LB)	PsO	Retrospective	LB (31 patients before and after, 86 only after, 51 only before treatment)	8/31 (25.8%) fibrosis, 3/31 (9.6%) cirrhosis
Zachariae H, 1991^20^	132	PsO (62), PsA (70)	Retrospective	LB	42 (31.8%) fibrosis or cirrhosis
Themido R, 1992^38^	30	PsO	Retrospective	LB (before and after treatment)	15 (50%) fibrosis, 3 (10%) cirrhosis
Nohlgård C, 1993^21^	26 (43 LB)	PsO	Prospective (??)	LB	25 (58%) fibrosis 4 (9.3%) cirrhosis
Boffa MJ, 1995^22^	49	PsO	Restrospective	LB (at least 2 under continued treatment)	11 (22.4%) fibrosis on first and 10 (20.4%) on sequential biopsy 0 cirrhosis
Malatjalian DA, 1996^23^	104	PsO	Retrospective	LB (baseline and annual follow-up)	21 (20%) fibrosis, 3 (2.9%) cirrhosis
Grismer L, 2001^24^	17 (21 LB)	PsA	Prospective	LB (3 pretreatment)	9 (50%) fibrosis 0 cirrhosis
Zachariae H, 2001^25^	70 with no fibrosis at baseline	PsO	Retrospective	LB	4/70 (5.7%) fibrosis under treatment
Wollina U, 2001^26^	104	PsO and PsA	Retrospective	Biochemical, Ultrasound, LB if abnormal	1/12 (8.3%) cirrhosis in LB, 1/104 (0.9%) cirrhosis in cohort
Rosenberg P, 2007^46^	71 (169 LB)	PsO	Retrospective	LB	51 (71%) fibrosis, 3 (4.2%) cirrhosis
Lindsay K, 2009^2^7	54	PsO (7) PsA (47)	Prospective	LB	11 (20%) fibrosis 0 cirrhosis
Laharie D, 2010^28^	111	PsO	Prospective	TE (LB if high TE value)	12 (10.1%) with severe fibrosis
Barbero-Villares A, 2011^30^	53 (18 psoriasis)	RA, PsO, IBD	Prospective	TE	3 (5.6%) fibrosis 1 (1.8%) cirrhosis
van der Voort EA, 2016^31^	74	PsO	Prospective	TE	6 (8,1%) advanced fibrosis
Talme T, 2017^32^	169	PsO	Prospective	TE	15/47 (31.9%) mild fibrosis 3/47 (6.4%) severe fibrosis [receiving MTX<24months] 46/122 (37.7%) mild fibrosis and 11/122 (9%) severe fibrosis when receiving MTX >24 months
Garcia D, 2019^33^	43	RA, PsA	Prospective	ALT, AST – LB when laboratory results abnormal	3 (7%) fibrosis 0 cirrhosis
Maybury CM, 2019^34^	333	PsO	Prospective (??)	TE	47 (14.1%) advanced fibrosis
Neema S, 2020^35^	82	PsO	Prospective	TE	23 (28%) fibrosis

**Abbreviations:** PsO: Psoriasis; PsA: Psoriatic arthritis; RA: Rheumatoid Arthritis; SLE: Systemic Lupus Erythematosus; MTX: Methotrexate; LB: Liver Biopsy; TE: Transient Elastography.

### Studies for psoriasis

For psoriasis, in one of the largest studies by Zacharie et al. in 1980, 764 liver biopsies (LB) before and after MTX treatment were performed in 328 individuals with psoriasis; 25.6% of patients developed liver cirrhosis 5 years after methotrexate treatment. However, performing subsequent liver biopsies in some of these (n=14), the authors showed that cirrhosis did not progress in most cirrhotic patients, even though they continued methotrexate.^41^ It should be mentioned though that no data concerning diabetes mellitus (DM), hyperlipidaemia or body-mass index (BMI) were available. In another large study by Maybury et al. in 2019, that included 333 patients with psoriasis, 14.1% of them showed advanced fibrosis using transient elastography (TE). In this study central obesity, insulin resistance and active psoriasis but not MTX exposure were predisposing factors for advanced fibrosis.^34^

### Studies for PsA

As far as PsA patients are concerned, in the largest cohort of them, including 70 PsA, 60 psoriasis patients and 39 controls, 18 of the 70 PsA patients (25.7%) had liver fibrosis or cirrhosis; however, no information about the severity of liver fibrosis or the number of cirrhotic patients is given.^20^ In the only study that includes almost exclusively PsA patients, with 47 PsA and only 7 patients with psoriasis and no articular involvement, 13% of the patients had early inflammation or fatty changes in LB, while 20% had liver fibrosis^27^; however, no grade 3 or 4 fibrosis was found. In both studies, no correlation between MTX use and liver fibrosis was noted.

### Data from meta-analyses

A couple of meta-analyses have also been conducted, trying to clarify the association between MTX and hepatotoxicity.^13^ In one meta-analysis by O’Keefe et al in 1991, 15 studies including patients with RA or PsO were analyzed. According to this meta-analysis, progression of liver disease was associated with MTX cumulative dose. Also, psoriatic patients under MTX treatment, compared to those with RA, had a higher probability of developing liver damage. Importantly, alcohol abuse was identified as a risk factor for liver-damage severity and progression.^13^ In another meta-analysis by Maybury et al. made in 2014,^48^ eight observational studies and a total population of 429 patients with PsO were included. MTX treatment appeared to contribute to any liver fibrosis and cirrhosis with a pooled risk difference of 0.22 (95% CI 0.04–0.41) and 0.04 (95% CI 0.02–0.07) respectively. No other risk factors like DM, high BMI value or alcohol abuse were found to contribute to liver fibrosis. However, the quality of the included studies was deemed to be weak by the authors.

Collectively, one could say that fibrosis and/or fibrosis is present in about 15–25% of patients with psoriasis or PsA. MTX, along with other risk factors, like alcohol abuse, DM, and obesity seem to act synergistically promoting liver damage. It is impossible to speculate though to which extent each factor contributes.

## DISCUSSION

We present a patient with PsA under long-term MTX treatment and diabetes mellitus that was admitted to our department with decompensated liver cirrhosis.

PsA is a chronic inflammatory arthritis associated with psoriasis accompanied by a variety of other clinical manifestations. Psoriasis and PsA are strongly associated with clinical features of metabolic syndrome (MetS), including insulin resistance, central obesity, elevated blood pressure, atherogenic dyslipidaemia, and non-alcoholic fatty liver disease.^49^ The prevalence of MetS and its individual components is higher in PsA patients compared to general population and patients with other rheumatic diseases.^50–53^ For instance, strong epidemiological data from cross-sectional studies correlate total cholesterol levels and body mass index (BMI) with joint and skin disease activity, thus implying a negative impact of MetS in achieving low disease activity and good clinical response in PsA patients.^54–56^

PsA and MetS share common pathophysiological pathways; endothelial dysfunction, dysregulation of innate immunity and increased pro-inflammatory cytokine production are a few of the mechanisms implicated.^57–59^ Adipokines are also increasingly recognized as important players in the interplay between PsA and MetS; they also play a role in the development of liver fibrosis/cirrhosis.^60–65^

In our patient, both NASH and MTX-induced cirrhosis could explain the clinical course. Histologically, MTX hepatotoxicity includes macrovesicular steatosis, ballooning degeneration and fibrosis, features also characteristic of non-alcoholic steatohepatitis. A MTX-related autoimmune hepatitis-like pattern showing portal and periportal interface inflammation has also been reported. Hepatocyte nuclear pleomorphism, hyperchromasia, and vacuolation are considered MTX-specific findings; however, they are not invariably present.^66^ In our case, the combination of histology findings with the clinical features make the diagnosis of MTX-related cirrhosis likely. In fact, we and other investigators believe that drugs work synergistically with other risk factors like diabetes, contributing to development and progression of liver injury. In our patient, despite the fact that glycaemic control was adequate for the last 5 years, diabetes mellitus most probably acted as an aggravating factor of MTX-related liver injury with the final outcome being cirrhosis.

We acknowledge that our case-based review has certain limitations. First, we cannot exclude the possibility that a degree of liver damage pre-existed in our patient, nor that NASH could be the major cause of his liver damage. Besides, as mentioned, there is a degree of overlap between NASH and MTX-related liver injury, at a histological level. The point of our study was to highlight and discuss the possible aggravating role of MTX in liver damage, especially when other risk factors concur. Furthermore, our review was not systematic; therefore, it was registered. Also, only two major databases were searched. In this context, some studies might have been missed. We expect however, that most of them would have been published in PubMed or Scopus.

It is unfortunate that only limited data exist, examining the level, prevalence, and contributing risk factors for liver damage in PsA. This possibly owes to the heterogeneity of the disease as well as to the availability of different techniques used for liver damage assessment (eg, liver biopsies, elastography). Apparently, more studies, of prospective nature and specifically designed for this purpose are needed.

In conclusion, most researchers agree that MTX by itself seldom leads to significant liver fibrosis, however one should consider methotrexate-induced cirrhosis when a patient has comorbidities, especially chronic hepatitis, diabetes mellitus, alcohol overconsumption or hypertriglyceridemia. It is of question, how often and with which tools these patients should be followed-up.
